# Thermodynamic Analysis of a Hybrid Power System Combining Kalina Cycle with Liquid Air Energy Storage

**DOI:** 10.3390/e21030220

**Published:** 2019-02-26

**Authors:** Tong Zhang, Xuelin Zhang, Xiaodai Xue, Guohua Wang, Shengwei Mei

**Affiliations:** 1 State Key Laboratory of Control and Simulation of Power System and Generation Equipment, Department of Electrical Engineering, Tsinghua University, Beijing 100084, China; 2 School of QiDi (TUS) Renewable Energy, Qinghai University, Xining 810016, China; 3 China Salt Jintan Chemical Co., Ltd., Changzhou 213200, China

**Keywords:** liquid air energy storage, Kalina cycle, heat recovery, thermodynamic analysis

## Abstract

Liquid air energy storage (LAES) is a promising energy storage technology in consuming renewable energy and electricity grid management. In the baseline LAES (B-LAES), the compression heat is only utilized in heating the inlet air of turbines, and a large amount of compression heat is surplus, leading to a low round-trip efficiency (RTE). In this paper, an integrated energy system based on LAES and the Kalina cycle (KC), called KC-LAES, is proposed and analyzed. In the proposed system, the surplus compression heat is utilized to drive a KC system to generate additional electricity in the discharging process. An energetic model is developed to evaluate the performance of the KC and the KC-LAES. In the analysis of the KC subsystem, the calculation results show that the evaporating temperature has less influence on the performance of the KC-LAES system than the B-LAES system, and the optimal working fluid concentration and operating pressure are 85% and 12 MPa, respectively. For the KC-LAES, the calculation results indicate that the introduction of the KC notably improves the compression heat utilization ratio of the LAES, thereby improving the RTE. With a liquefaction pressure value of eight MPa and an expansion pressure value of four MPa, the RTE of the KC-LAES is 57.18%, while that of the B-LAES is 52.16%.

## 1. Introduction

Large-scale energy storage is an effective solution for improving and expanding renewable energy systems. It can also be used for the storage of electrical energy and for grid load shifting. Currently, pumped hydro energy storage (PHES) and compressed air energy storage (CAES) are the major large-scale energy storage technologies. PHES is well-developed and efficient, but it is restricted by geological characteristics. Small-scale CAES systems usually use a high-pressure container as the air storage tank [1], which increases the investment cost and limits the install capacity. In large-scale CAES systems, salt caverns and underground mines can be used for storing the high-pressure air [2], but here again, the locations of the plants are restricted by geological characteristics.

Apart from PHES and CAES, another promising solution for large-scale energy storage is liquid air energy storage (LAES), which has the notable advantages of high energy-storage density and no geological constraints. The concept of storing energy in liquid air was first proposed by Smith in 1977 [3], and Highview Power Storage Ltd. designed and established the world’s first LAES pilot plant (350 kW/2.5 MWh) [4]. Since then, many researchers have studied the performance of standalone LAES and integrated LAES systems with various configurations. Ameel et al. [5] investigated a thermodynamic cycle for energy storage using liquid air, and a maximum round-trip efficiency (RTE) of 43.3% was attained. Sciacovelli et al. [6] studied a standalone LAES system with packed bed cold energy storage. The dynamic characteristics of the system were analyzed, and an RTE of 50% was obtained. Xue et al. [7] presented a thermodynamic analysis of a standalone LAES system, and the influence of key parameters was discussed. Guizzi et al. [8] investigated an LAES system with cryogenic liquid cold energy storage and assessed its efficiency. An RTE in the range of 54–55% could be obtained with reasonable and conservative design parameters. Howe et al. [9] presented an energy and exergy analysis for a combined building-scale LAES system. Their analytical approach can be applied to other LAES configurations to identify optimal operating parameters. Tafone et al. [10] studied an LAES system as a cooling application in hot climates, and an RTE of 45% was obtained.

In the study of integrated LAES systems, Li et al. [11] proposed a hybrid system, integrating LAES with nuclear power plants and obtaining a high RTE of 70%. Zhang et al. [12] introduced the cold energy of liquefied natural gas (LNG) into the liquefaction process of LAES, and a higher electrical energy storage efficiency was obtained. Antonelli et al. [13] analyzed the potential and challenges of hybrid power plants based on LAES; the performance of possible configurations was analyzed and compared. Al-Zareer et al. [14] studied hybrid LAES systems for district heating and cooling. Brayton, Rankine, and absorption cooling cycles were introduced into the system, and an RTE of about 70% was obtained.

In baseline LAES (B-LAES), the compression heat is surplus because of the low liquefaction ratio. Therefore, the effective utilization of compression heat has a significant influence on the RTE of an LAES system. She et al. [15] introduced an organic Rankine cycle (ORC) system driven by the surplus compression heat into an LAES system to improve its RTE. In a subsequent study, Peng et al. [16] compared two configurations of ORC systems with different cold sources: ambient and a low-temperature cold source obtained through an absorption refrigeration cycle. The results indicated that the introduction of an ORC can effectively improve the performance of the system, and the LAES combined with the ambient ORC system had the better performance. In order to further improve the RTE of LAES, Tafone et al. [17,18] studied and compared different integrated LAES systems consisting of an ORC and an absorption chiller. The results showed that such integrated energy systems can significantly improve efficiency and reduce the payback period.

Apart from the ORC system, another representative technology for utilizing waste heat to generate electricity is the Kalina cycle (KC). The KC used ammonia–water as the working fluid to realize a good temperature match between the heat source and working fluid due to the variable boiling temperature of ammonia–water [19,20]. Numerous investigations have been carried out to study the performance of the KC from the perspective of the first and second law of thermodynamics [21,22]. Moreover, the KC also has been considered as both the bottom and topping cycle in the integrated systems. For example, Zhao et al. [23] presented a thermodynamic analysis of an integrated energy system based on CAES and KC. In another study, Li et al. [24] compared the performance of KC and ORC in recovering the residual heat of CAES. In this paper, a novel integrated energy system based on LAES and KC (KC-LAES) is proposed and studied. In the discharging process of the proposed system, the stored compression heat is first used to heat the inlet air to the air turbines; then, the surplus portion is used to drive the KC subsystem to generate additional electricity. A mathematical model is developed to analyze the performance of the KC and the integrated energy system. In the analysis of the KC, the influence of the basic concentration of ammonia–water, the evaporating temperature, and the operating pressure is discussed. In the analysis of the KC-LAES, the influence of the liquefaction and expansion pressure is studied. Finally, the calculation results of the KC-LAES with typical operating parameters are presented and discussed.

## 2. System Description

**Figure 1:**
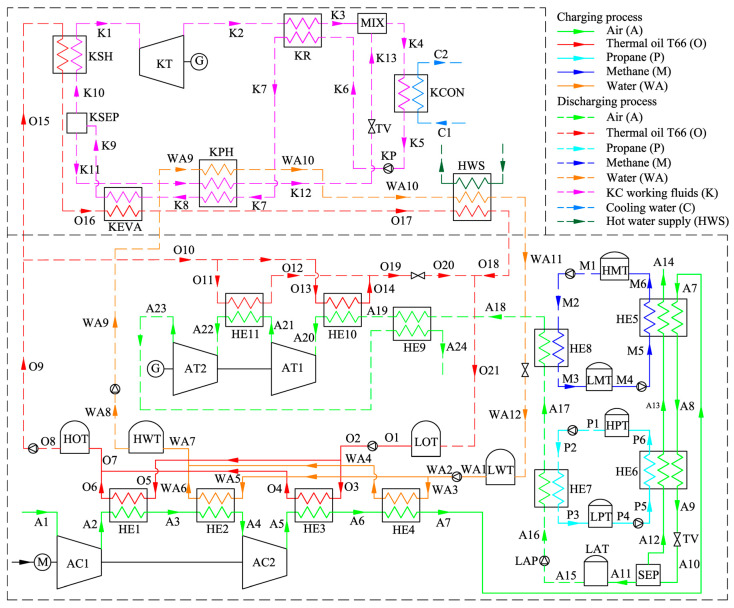
Schematic of the Kalina cycle liquid air energy storage (KC-LAES) system.

**Figure 2:**
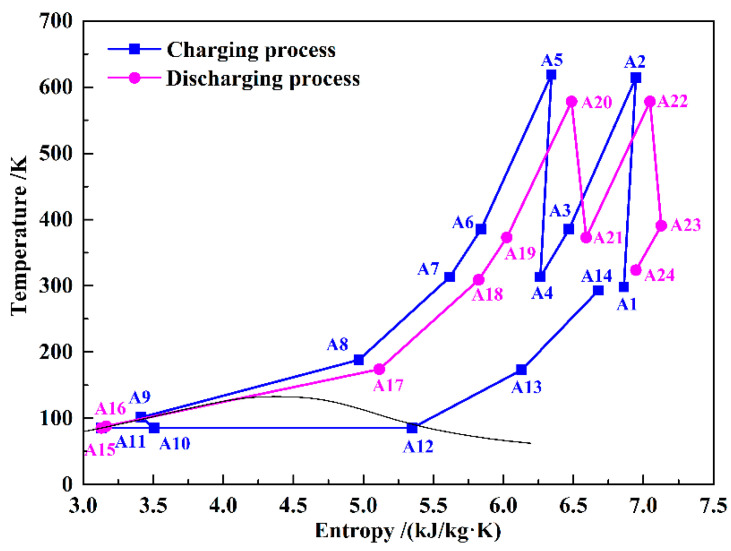
T–s diagram of the baseline liquid air energy storage (B-LAES) system.

Figure 1 shows the schematic of the proposed KC-LAES. In order to clearly describe and analyze the system, all the streams have been numbered. The bottom part of Figure 1 is a typical B-LAES with cryogenic liquid cold energy storage, and Figure 2 shows its T–s diagram. In the charging process, the air is compressed to a high-pressure state (A7), and then cooled to a liquid state (A9). The high and low temperature compression heat generated in the compression process is stored in thermal oil and water, respectively. The cold energy utilized in cooling the compressed air is harvested in the discharging process. Then, a vapor–liquid mixture (A10) is obtained through an expansion process in the throttle valve (TV). After separation, the liquid air (A11) is stored in the liquid air tank (LAT), and the gaseous air (A12) flows black to cool the compressed air. In the discharging process, the liquid air is first pumped to a high-pressure state (A16), and then flows through a two-stage heat exchanger to be gasified. During the gasification process, the cold energy of the liquid air is stored in methane or propane, depending on the temperature, and then utilized in the liquefaction process in the next cycle. Before flowing into the turbines, the air is heated by the high-temperature compression heat stored in the thermal oil. A regenerator is introduced to reduce the temperature of the exhausted gas. 

**Figure 3:**
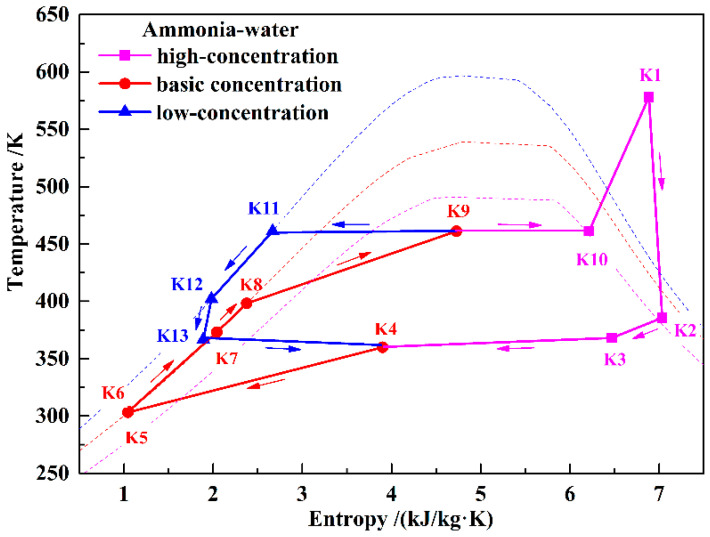
T–s diagram of the KC subsystem.

The upper part of Figure 1 shows a schematic of the KC. In order to simplify the system, a simple regenerative KC is assumed in this paper. Figure 3 shows the T–s diagram of the KC system. During the discharging process, the KC turbine (KT) and the air turbines work simultaneously to generate electricity. Ammonia–water with a certain concentration has been chosen as the working fluid. In the KC system, the low-temperature basic concentration ammonia–water (BCAW) is first pumped to a high-pressure state (K6). Then, before flowing into the KC evaporator (KEVA), the BCAW is gradually heated by the exhausted gas in the KC regenerator (KR) and the low-concentration ammonia–water (LCAW) in the KC preheater (KPH). The low-temperature compression heat stored in water can also be used to preheat the BCAW in the KPH if the heat of the LCAW is insufficient. In the KEVA, the BCAW is heated by the high-temperature compression heat stored in thermal oil, and a liquid–vapor mixture is obtained (K9). In the KC separator (KSEP), the mixture is separated into a high-concentration ammonia–water (HCAW) stream (K10) and an LCAW stream (K11). The HCAW is then heated to a superheated state (K1) by the thermal oil and expanded in the KT to generate electricity. The expanded gas (K2) flows through the KR to heat the BCAW and mixes with the throttled LCAW in the mixer (MIX). Finally, the liquid–vapor mixture (K4) is cooled to a liquid state (K5) by the cooling water in the KC condenser (KCON). Before flowing back to the low-temperature storage tank, the thermal oil and water are cooled, and a hot water supply can be obtained. In this paper, only the power generation is considered in analyzing the performance of the proposed system; the heating supply is neglected.

## 3. Thermodynamic Analysis Model

### 3.1. Basic Assumptions

The Aspen HYSYS^®^ software was used to analyze the performance of the proposed system. The classical Peng–Robinson equation of state was selected as the property package, and the properties of the working fluids were selected from the HYSYS source database. Following are the main assumptions made in this study:

1. The systems work in the steady state, and the durations of the charging and discharging processes are the same, i.e., four hours.
2. The ambient air is composed of 78% nitrogen, 20.93% oxygen, 0.03% carbon dioxide, 0.09% water, and 0.94% argon.
3. The temperature difference at the pinch point is two K in the heat exchangers that have phase changes and five K in the other heat exchangers.
4. The heat exchangers are countercurrent flow types with a reasonable pressure drop and no heat leakage.
5. The temperature decrease of the high-temperature compression heat storage tank in a cycle is two K.

### 3.2. Energy Analysis Model

The RTE is an important indicator for evaluating the performance of an LAES system. In this study, the RTE is defined as the power generation in the discharging process divided by the power consumption in the charging process. With the basic assumptions of the study, the durations of the charging and discharging processes are the same. Therefore, the influence of time can be neglected in the definition of the RTE. The RTE for B-LAES can be expressed as:

(1) $$\eta_{\mathrm{RTE},\mathrm{B-LAES}}=\frac{W_{\mathrm{AT}1}+W_{\mathrm{AT}2}}{W_{\mathrm{AC}1}+W_{\mathrm{AC}2}+W_{\mathrm{LAP}}}$$

where *W*_AC1_ and *W*_AC2_ represent the power consumption of the first-stage and second-stage compressors, respectively; *W*_LAP_ represents the power consumption of the liquid air pump (LAP) (the power consumed by the other pumps is neglected because of their low values); and *W*_AT1_ and *W*_AT2_ represent the power generation of the first-stage and second-stage turbines, respectively.

The RTE of the KC-LAES can be expressed as:

(2) $$\eta_{\mathrm{RTE},\mathrm{KC-LAES}}=\frac{W_{\mathrm{AT}1}+W_{\mathrm{AT}2}+W_{\mathrm{KT}}}{W_{\mathrm{AC}1}+W_{\mathrm{AC}2}+W_{\mathrm{LAP}}+W_{\mathrm{KP}}}$$

where *W*_KT_ represents the power generation of the KT, *W*_KP_ represents the power consumption of the KC pump (KP), and the other parameters are the same as those given for Equation (1).

The compression heat utilization ratio *γ*_CH_ is defined as the compression heat utilized in the discharging process divided by the compression heat stored in the charging process. Therefore, the *γ*_CH_ for B-LAES and the KC-LAES can be expressed as:

(3) $$\gamma_{\mathrm{CH},\mathrm{B-LAES}}=\frac{m_{O10}\left(h_{O10}-h_{O19}\right)}{m_{O2}\left(h_{O7}-h_{O2}\right)+m_{\mathrm{WA}7}\left(h_{\mathrm{WA}7}-h_{\mathrm{WA}2}\right)}$$

(4) $$\gamma_{\mathrm{CH},\mathrm{KC-LAES}}=\frac{m_{O9}\left(h_{O9}-h_{O14}\right)+m_{O15}\left(h_{O15}-h_{O17}\right)+m_{\mathrm{WA}9}\left(h_{\mathrm{WA}9}-h_{\mathrm{WA}10}\right)}{m_{O2}\left(h_{O7}-h_{O2}\right)+m_{\mathrm{WA}7}\left(h_{\mathrm{WA}7}-h_{\mathrm{WA}2}\right)}$$

where *m* and *h* represent the mass flow rate and specific enthalpy, respectively, and the subscripts represent the states as shown in Figure 1.

For the KC system, the efficiency and exergy efficiency can be expressed respectively as:

(5) $$\eta_{\mathrm{KC}}=\frac{W_{\mathrm{net},\mathrm{KC}}}{Q_{\mathrm{CH},\mathrm{KC}}}=\frac{W_{\mathrm{KT}}-W_{\mathrm{KP}}}{m_{O15}\left(h_{O15}-h_{O17}\right)+m_{\mathrm{WA}9}\left(h_{\mathrm{WA}9}-h_{\mathrm{WA}10}\right)}$$

(6) $$\eta_{\mathrm{ex},\mathrm{KC}}=\frac{W_{\mathrm{net},\mathrm{KC}}}{E_{\mathrm{CH},\mathrm{KC}}}=\frac{W_{\mathrm{KT}}-W_{\mathrm{KP}}}{m_{O15}\left(ex_{O15}-ex_{O17}\right)+m_{\mathrm{WA}9}\left(ex_{\mathrm{WA}9}-ex_{\mathrm{WA}10}\right)}$$

where *Q*_CH_ and *E*_CH_ represent the energy and exergy of the utilized compression heat, respectively; *W*_net_ represents the net power generation; and *ex* represents the specific exergy.

The improvement in the RTE can be expressed as:

(7) $$\eta_{\mathrm{RTE},\mathrm{imp}}=\eta_{\mathrm{RTE},\mathrm{KC-LAES}}-\eta_{\mathrm{RTE},\mathrm{B-LAES}}$$

## 4. Results and Discussion

In this section, the performance of the proposed KC-LAES is presented and discussed. Table 1 lists the basic design parameters of the system. In the proposed system, the air compression and expansion are both two-stage processes, and the adiabatic efficiencies of the air compressors and turbines have been selected as 85%. The pressure and temperature of the inlet air of the first-stage compressor are 0.101 MPa and 298.15 K, respectively. The mass flow rate of the air is fixed at 33.33 kg/s, and the storage pressure of the liquid air has been chosen as 0.2 MPa. In the KC subsystem, the KT is a single-stage turbine, and its adiabatic efficiency has likewise been selected as 85%. In the proposed system, the adiabatic efficiencies of all the pumps have been selected as 75%. In the discussions presented in this section, the pressure of the outlet air of the second-stage compressor (A5) is defined as the liquefaction pressure, and the pressure of the outlet air of the liquid air pump (LAP) is defined as the expansion pressure (A16). In the KC subsystem, the temperature and pressure of the outlet of the KEVA (K9) are defined as the evaporating temperature and operating pressure, respectively.

### 4.1. Analysis of the KC Subsystem

In the proposed system, the compression heat that can be utilized in the KC varies according to the operating parameters of the LAES. Table 2 lists the compression heat parameters utilized in the KC system under typical operating parameters (liquefaction pressure of eight MPa and expansion pressure of four MPa). With this operating condition, the total mass flow rate of thermal oil and hot water in the discharging process is 36.46 kg/s and 16.12 kg/s, respectively. A large amount of thermal oil is utilized in heating the inlet air of air turbines, and the mass flow rate is 26.88 kg/s. The surplus part can be utilized in driving the KC, and the mass flow rate is 9.58 kg/s. The hot water is not utilized in the discharging process of LAES, and the mass flow rate that can be utilized in KC is 16.12 kg/s. Besides, the inlet temperatures of thermal oil and hot water are 584.65 K and 380.15 K, respectively. As the temperature of the thermal oil is fixed, the superheated temperature of the HCAW is also constant. The influence of the basic ammonia–water concentration (*x*_BCAW_), evaporating temperature (*T*_EVA_), and operating pressure (*P*_KC_) on the performance of the KC is analyzed in this section. The power generation and efficiencies for each calculation case are also presented.

**Figure 4:**
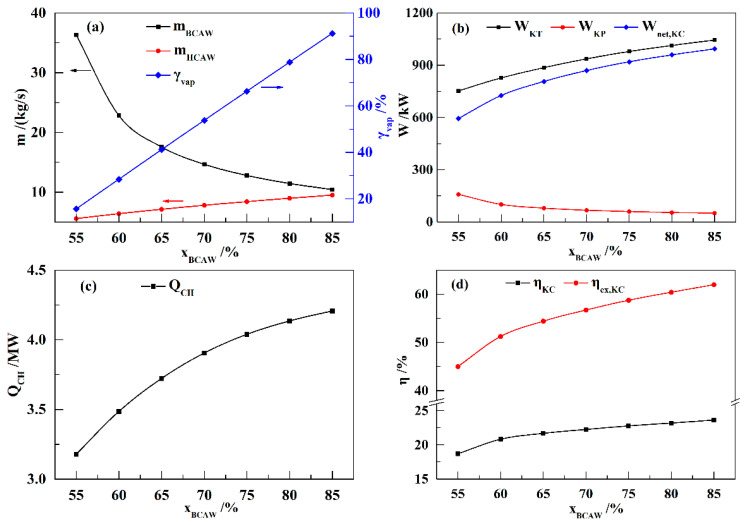
Influence of *x*_BCAW_ on the performance of the KC (*T*_EVA_ = 460 K, *P*_EVA_ = 9 MPa): (**a**) *m*_BCAW_, *m*_HCAW_, and *γ*_vap_; (**b**) *W*_KT_, *W*_KP_, and *W*_net,KC_; (**c**) *Q*_CH_; (**d**) *η*_KC_ and *η*_ex,KC_.

Figure 4 shows the influence of *x*_BCAW_ on the performance of the KC subsystem. In the KC, the mass flow rate of BCAW (*m*_BCAW_) is the maximum flow rate, and it primarily affects the power consumption of the KP. The mass flow rate of HCAW (*m*_HCAW_)—determined by *m*_BCAW_ and the vapor friction of K9 (*γ*_vap_)—and the *P*_KC_ value are the main factors that influence the power generation of the KT. As shown in Figure 4a, *m*_BCAW_ decreases with increasing *x*_BCAW_, but *γ*_vap_ increases linearly. Therefore, *m*_HCAW_ increases with increasing *x*_BCAW_. Since *m*_BCAW_ decreases and *m*_HCAW_ increases with increasing *x*_BCAW_, the power generation of the KT (*W*_KT_) increases, and the power consumption of the KP (*W*_KP_) decreases. Therefore, the net power generation of the KC (*W*_net,KC_) increases with the increasing *x*_BCAW_, as shown in Figure 4b. Meanwhile, the compression heat utilized in the KC (*Q*_CH_) also increases with increasing *x*_BCAW_ (Figure 4c). As shown in Figure 4d, the efficiency (*η*_KC_) and exergy efficiency (*η*_ex,KC_) increase with increasing *x*_BCAW_, but the increment gradually decreases. The results with higher *x*_BCAW_ are not presented, because the calculations diverge when *x*_BCAW_ exceeds 85%.

**Figure 5:**
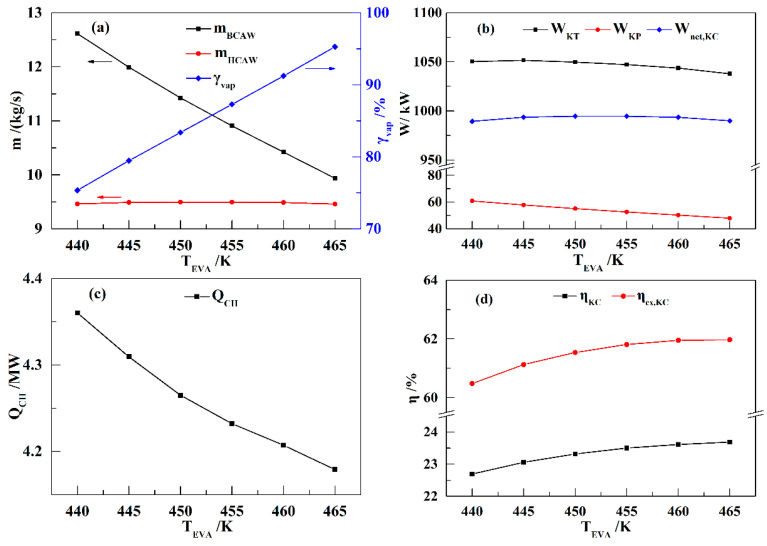
Influence of *T*_EVA_ on the performance of the KC (*x*_BCAW_ = 85%, *P*_EVA_ = nine MPa): (**a**) *m*_BCAW_, *m*_HCAW_, and *γ*_vap_; (**b**) *W*_KT_, *W*_KP_, and *W*_net,KC_; (**c**) *Q*_CH_; (**d**) *η*_KC_ and *η*_ex,KC_.

Figure 5 shows the influence of *T*_EVA_ on the performance of the KC subsystem. Generally, *T*_EVA_ has a slight influence on the performance of the KC. As shown in Figure 5a, *m*_BCAW_ decreases linearly and *γ*_vap_ increases linearly with increasing *T*_EVA_ values. Therefore, *m*_HCAW_ remains nearly constant. Correspondingly, *W*_KT_ and *W*_net,KC_ present little variation with increasing *T*_EVA_ values (Figure 5b). As *Q*_CH_ decreases with increasing *T*_EVA_ values (Figure 5c), the *η*_KC_ and *η*_ex,KC_ values increase slightly with increasing *T*_EVA_ values, as shown in Figure 5d.

**Figure 6:**
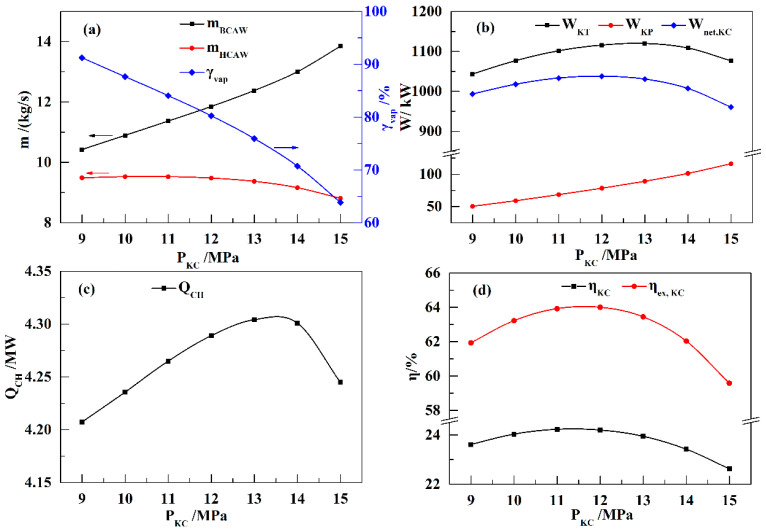
Influence of *P*_KC_ on the performance of the KC (*x*_BCAW_ = 85%, *T*_EVA_ = 460 K): (**a**) *m*_BCAW_, *m*_HCAW_, and *γ*_vap_; (**b**) *W*_KT_, *W*_KP_, and *W*_net,KC_; (**c**) *Q*_CH_; (**d**) *η*_KC_ and *η*_ex,KC_.

Figure 6 shows the influence of *P*_KC_ on the performance of the KC subsystem. With increasing *P*_KC_ values, *m*_BCAW_ increases, and *γ*_vap_ decreases rapidly. The *m*_HCAW_ value remains nearly constant with the initial increase in *P*_KC_, but presents a decrease when *P*_KC_ exceeds 12 MPa, as shown in Figure 6a. Therefore, *W*_KT_ first increases and then decreases with the increase in *P*_KC_, as shown in Figure 6b. The value of *W*_KP_ increases with increasing *P*_KC_ values, and *W*_net,KC_ presents a trend similar to that of *W*_KT_. As shown in Figure 6c, *Q*_CH_ also increases at first, and then decreases with the increase in *P*_KC_. Therefore, an optimal *P*_KC_ value of 11–12 MPa is seen to achieve the highest *η*_KC_ and *η*_ex,KC_ values, as shown in Figure 6d.

### 4.2. Analysis of the KC-LAES

**Figure 7:**
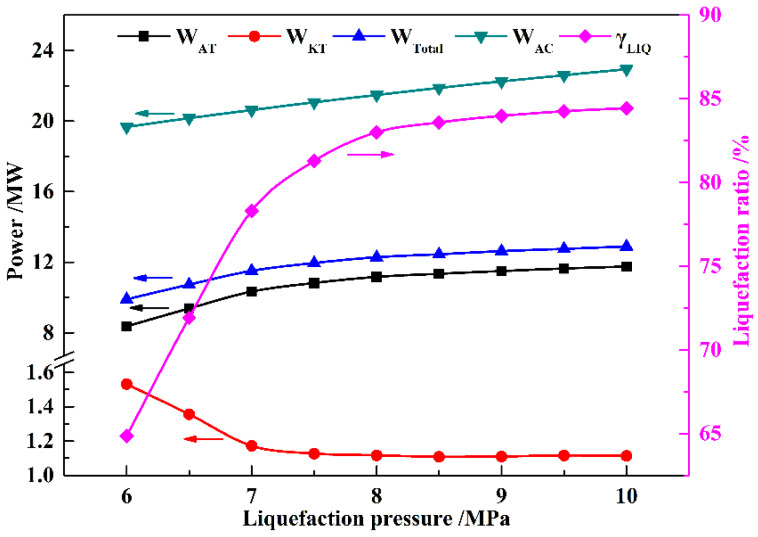
Influence of liquefaction pressure on *W*_AT_, *W*_KT_, *W*_Total_, *W*_AC_, and *γ*_LIQ_ (with an expansion pressure value of four MPa).

The influence of the key parameters on the performance of the proposed KC-LAES is discussed in this section. In the calculations for this section, the operating parameters of the KC system are fixed (*x*_BCAW_ of 85%, *T*_EVA_ of 460 K, and *P*_KC_ of 12 MPa). Figure 7 shows the influence of liquefaction pressure on the power generation and consumption of the proposed system. Compared with the power consumption of air compressors, the power consumption of pumps is low. Therefore, the power consumption of the pumps is not shown in the figure. With the increasing liquefaction pressure, the liquefaction ratio (*γ*_LIQ_) increases, but beyond a pressure of eight MPa, the ratio increases only a little. The increasing *γ*_LIQ_ means an increase in the mass flow rate of the air expanded in the air turbines. As the expansion pressure is fixed, the power generation of the air turbines (*W*_AT_) presents a trend similar to that of *γ*_LIQ_. Moreover, the increasing *γ*_LIQ_ means that more compression heat utilized in heating the inlet air of the air turbines, and less is utilized in the KC subsystem. Thus, *W*_KT_ decreases with the increasing liquefaction pressure, and remains nearly constant beyond eight MPa. The value of *W*_KT_ is much smaller than that of *W*_AT_. Therefore, the trend shown by *W*_Total_ is similar to that of *W*_KT_. 

**Figure 8:**
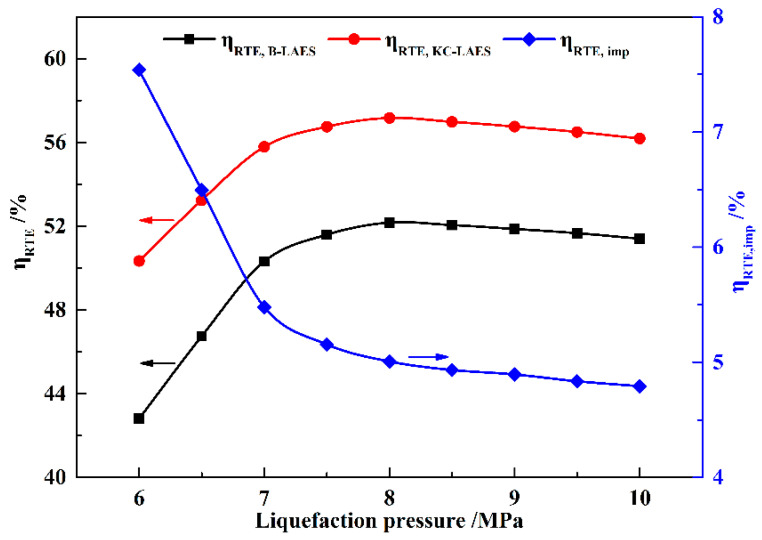
Influence of liquefaction pressure on round-trip efficiencies (RTEs) (with an expansion pressure value of four MPa).

Figure 8 shows the influence of the liquefaction pressure on the RTEs of B-LAES and the KC-LAES. As shown in Figure 7, the power consumption of the air compressors (*W*_AC_) increases linearly with increasing liquefaction pressure. However, the increment of *W*_Total_ decreases as the liquefaction pressure increase. Therefore, the RTEs of B-LAES and the KC-LAES first increase with increasing liquefaction pressure and then present a slight decrease when the liquefaction pressure exceeds eight MPa. The RTE of the KC-LAES is improved by 4.8–7.54% over that of B-LAES in the liquefaction pressure range of six to 10 MPa, owing to the additional electricity from the KC subsystem. Since *W*_KT_ decreases with increasing liquefaction pressure, *η*_RTE,imp_ also decreases with increasing liquefaction pressure.

**Figure 9:**
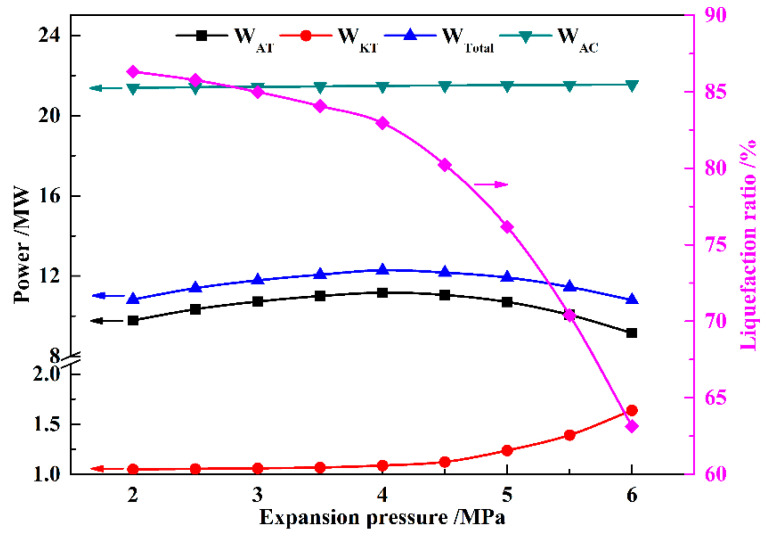
Influence of expansion pressure on *W*_AT_, *W*_KT_, *W*_Total_, *W*_AC_, and *γ*_LIQ_ (with a liquefaction pressure value of eight MPa).

The influence of the expansion pressure on the power generation and consumption of the proposed system is shown in Figure 9. Since the liquefaction pressure is fixed, *W*_AC_ remains essentially constant. With increasing expansion pressure, *γ*_LIQ_ decreases rapidly, and the mass flow rate of the air expanded in the air turbines also decreases. Therefore, *W*_AT_ and *W*_Total_ first increase and then decrease with the increasing expansion pressure. As *γ*_LIQ_ decreases, the compression heat utilized in the LAES decreases, and the part utilized in the KC subsystem correspondingly increases, leading to an increase in *W*_KT_, as shown in the figure.

**Figure 10:**
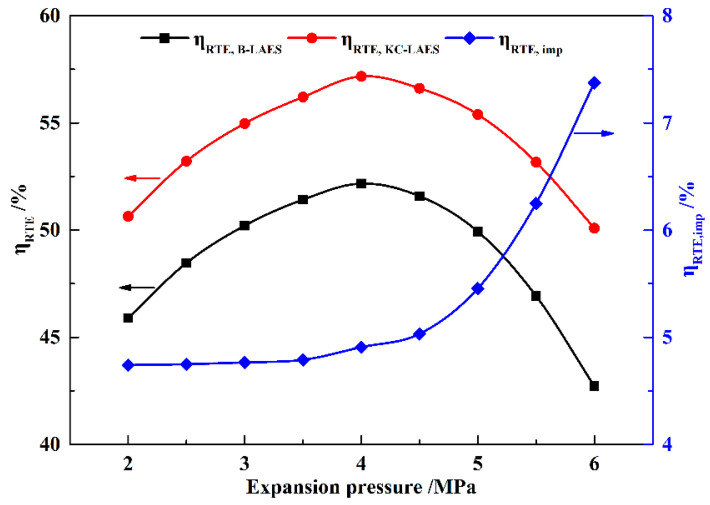
Influence of expansion pressure on RTEs (with a liquefaction pressure value of eight MPa).

Figure 10 shows the influence of the expansion pressure on the RTEs of B-LAES and the KC-LAES. As *W*_AC_ is essentially constant, the RTEs present trends similar to that of *W*_Total_, and an optimal expansion pressure value of four MPa is obtained. The value of *η*_RTE,imp_ increases markedly with the increase in expansion pressure because of the increase in *W*_KT_. The RTE of the KC-LAES is improved by 4.74–7.37% over that of B-LAES in the expansion pressure range of 2.0 to 6.0 MPa.

### 4.3. Performance of the KC-LAES with Typical Operating Conditions

The performance of the KC-LAES with typical operating conditions is presented in this section. Table 3 and Table 4 show the thermodynamic parameters of air, thermal oil, and water streams. As listed in Table 3, the liquefaction pressure (A5) is eight MPa, and the expansion pressure (A16) is four MPa. The mass flow rate of the inlet air of the compressor is fixed at 33.33 kg/s. After separation, the mass flow rates of the liquid and gaseous air are 27.67 kg/s and 5.66 kg/s, respectively. The temperature of the outlet air of AT1 and AT2 are 614.44 K and 618.4 K, respectively. Considering a reasonable temperature difference between hot and cold fluids in the heat exchangers, the storage temperatures of the thermal oil and water are 586.65 K and 380.15 K, respectively. In the basic assumptions, the temperature decrease of the high-temperature compression heat storage tank in a cycle is two K. Therefore, the temperatures of the outlet fluid from the high-temperature thermal oil tank (HOT) and the high-temperature water tank (HWT) are 584.65 K and 378.15 K, respectively. With the operating conditions given in this section, the mass flow rates of the thermal oil utilized in the air turbines and the KC subsystem are 26.88 kg/s and 9.58 kg/s, respectively. In the discharging process, the temperature of the inlet air to the air turbines has been chosen as 579.65 K. The temperatures of the expanded and exhausted air are 391.93 K and 324.68 K, respectively. In the KC of the presented case, the heat of the LCAW is sufficient to preheat the cold working fluid, and the compression heat stored in water is not necessary. Therefore, the thermodynamic parameter values for WA9 and WA10 are the same.

Table 5 lists the thermodynamic parameters of the ammonia–water streams in the KC subsystem. In addition to temperature, pressure, and mass flow rate, the concentration (*x*) and vapor fraction (***γ*_vap_**) of each stream are also presented. In the present case, the *x*_BCAW_ value has been chosen as 85%, and the calculated results for *x*_HCAW_ and *x*_LCAW_ are 90.94% and 61.26%, respectively. The *T*_EVA_ and *P*_KC_ values have been selected as 460 K and 12 MPa, respectively. Considering the minimum temperature difference of the KC superheater (KSH), the temperature of the inlet fluid to the KT has been chosen as 582.65 K. The *m*_BCAW_ and *m*_HCAW_ values are 3.25 kg/s and 2.60 kg/s, respectively. In the KCON, the liquid–vapor mixture is condensed by the cooling water, and the condensation pressure and temperature are 0.89 MPa and 300 K, respectively.

Table 6 shows a comparison of the calculation results for B-LAES and the KC-LAES with typical operating parameters. According to the calculation results, the performance of the system is significantly improved by the introduction of the KC. The utilization ratio of the compression heat is increased from 54.74% to 74.27%, and the RTE is correspondingly improved. With the same operating parameters, the RTEs of the B-LAES and the KC-LAES are 52.16% and 57.18%, respectively. Owing to the increase in the power generation, an energy storage density of 98.01 kWh/m^3^ is obtained in the KC-LAES, which is much higher than that of the B-LAES.

## 5. Conclusions

In this paper, a novel integrated system based on liquid air energy storage (LAES) and Kalina cycle (KC), called KC-LAES, has been proposed and analyzed. In baseline LAES (B-LAES), the compression heat is surplus because of the low liquefaction ratio. Therefore, a KC system is introduced into the LAES to utilize the surplus compression heat to generate additional electricity. An energetic model was developed to assess the performance of the proposed system. In the analysis of the KC, the influence of the working fluid concentration, evaporating temperature, and operating pressure was discussed. The power generation of the KC turbine, efficiency, and exergy efficiency for each calculation case was presented. According to the calculation results, the evaporating temperature has less influence on the performance of the KC, and the optimal working fluid concentration and operating pressure are 85% and 12 MPa, respectively. In the analysis of the KC-LAES, the influence of liquefaction and of expansion pressure was presented. The calculation results indicate that the introduction of the KC can notably improve the performance of LAES. The RTE of the KC-LAES is 57.18%, compared with that of B-LAES, 52.16%, with a liquefaction pressure value of eight MPa and an expansion pressure value of four MPa. 

## Figures and Tables

**Table 1:** Basic design parameters of the KC-LAES system. KT: KC turbine.

Parameters	Units	Values
Ambient pressure	MPa	0.101
Ambient temperature	K	298.15
Compression stage	—	2
Expansion stage	—	2
Air compression duration	h	4
Air expansion duration	h	4
Air flow rate of compressor	kg/s	33.33
Adiabatic efficiency of air compressors	%	85
Adiabatic efficiency of air turbines	%	85
Adiabatic efficiency of KT	%	85
Adiabatic efficiency of pumps	%	75
Storage pressure of liquid air	MPa	0.2

**Table 2:** Compression heat and cooling water parameters utilized in the KC system.

Parameters	Units	Values
Inlet temperature of thermal oil	K	584.65
Mass flow rate of thermal oil	kg/s	9.58
Inlet temperature of hot water	K	380.15
Mass flow rate of hot water	kg/s	16.12
Condensation temperature	K	300.15
Inlet temperature of cooling water	K	298.15
Outlet temperature of cooling water	K	303.15

**Table 3:** Thermodynamic parameters of air streams.

Stream	*T* (K)	*P* (MPa)	*m* (kg/s)	Stream	*T* (K)	*P* (MPa)	*m* (kg/s)
A1	298.15	0.10	33.33	A13	173.15	0.19	5.66
A2	614.44	0.987	33.33	A14	293.15	0.18	5.66
A3	385.15	0.967	33.33	A15	85.41	0.2	27.67
A4	313.15	0.947	33.33	A16	88.02	4	27.67
A5	618.40	8	33.33	A17	169.04	3.96	27.67
A6	385.15	7.92	33.33	A18	308.98	3.92	27.67
A7	313.15	7.84	33.33	A19	373.15	3.88	27.67
A8	188.15	7.76	33.33	A20	579.65	3.84	27.67
A9	101.35	7.68	33.33	A21	374.05	0.565	27.67
A10	85.41	0.2	33.33	A22	579.65	0.555	27.67
A11	85.41	0.2	27.67	A23	391.93	0.11	27.67
A12	85.41	0.19	5.66	A24	324.68	0.1	27.67

**Table 4:** Thermodynamic parameters of thermal oil and water streams.

Stream	*T* (K)	*P* (MPa)	*m* (kg/s)	Stream	*T* (K)	*P* (MPa)	*m* (kg/s)
O1	378.15	0.1	36.46	O18	378.15	0.1	9.58
O2	378.15	0.12	36.46	O19	378.15	0.14	26.88
O3	378.15	0.12	18.7	O20	378.15	0.1	26.88
O4	586.65	0.1	18.7	O21	378.15	0.1	36.46
O5	378.15	0.12	17.76	WA1	308.15	0.1	16.12
O6	586.65	0.1	17.76	WA2	308.15	0.2	16.12
O7	586.65	0.1	36.46	WA3	308.15	0.2	8.36
O8	584.65	0.1	36.46	WA4	380.15	0.18	8.36
O9	584.65	0.16	36.46	WA5	308.15	0.2	7.76
O10	584.65	0.16	26.88	WA6	380.15	0.18	7.76
O11	584.65	0.16	13.28	WA7	380.15	0.18	16.12
O12	378.15	0.14	13.28	WA8	378.15	0.18	16.12
O13	584.65	0.16	13.6	WA9	378.15	0.2	16.12
O14	378.15	0.14	13.6	WA10	378.15	0.2	16.12
O15	584.65	0.16	9.58	WA11	308.15	0.18	16.12
O16	534.35	0.14	9.58	WA12	308.15	0.1	16.12
O17	387.55	0.12	9.58				

**Table 5:** Thermodynamic parameters of ammonia–water streams.

Stream	*T* (K)	*P* (MPa)	*m* (kg/s)	*x* (%)	*γ*_vap_ (%)
K1	579.65	11.94	2.60	90.94	100
K2	369.88	0.9	2.60	90.94	97.46
K3	324.23	0.895	2.60	90.94	81.49
K4	324.69	0.895	3.25	85	68.88
K5	300	0.89	3.25	85	0
K6	303.26	12.36	3.25	85	0
K7	367.55	12.24	3.25	85	0
K8	385.5	12.12	3.25	85	0
K9	460	12	3.25	85	80.24
K10	460	12	2.60	90.94	100
K11	460	12	0.65	61.26	0
K12	369.65	11.94	0.65	61.26	0
K13	369.65	0.9	0.65	61.26	17.85

**Table 6:** Calculation results of the B-LAES and KC-LAES.

Parameters	Units	B-LAES	KC-LAES
Compressor power consumption	MW	21.22	21.22
Pump power consumption	MW	0.176	0.255
Air turbine power generation	MW	11.16	11.16
KT power generation	MW	—	1.12
Round-trip efficiency	%	52.16	57.18
Compression heat utilization ratio	%	54.74	74.27
Liquefaction ratio	%	83.01	83.01
Mass of stored liquid air	t	398.4	398.4
Volume of stored liquid air	m^3^	501.2	501.2
Specific consumption of liquid air	kWh/kg	0.2148	0.2156
Electricity energy storage density	kWh/m^3^	89.07	98.01

## Abbreviations

AC	air compressor
AT	air turbine
B-LAES	baseline LAES
BCAW	basic concentration ammonia–water
CAES	compressed air energy storage
HCAW	high-concentration ammonia–water
HE	heat exchanger
HMT	high-temperature methane tank
HOT	high-temperature thermal oil tank
HPT	high-temperature propane tank
HWS	hot water supply
HWT	high-temperature water tank
KC	Kalina cycle
KC-LAES	integrated energy system based on LAES and KC
KCON	KC condenser
KEVA	KC evaporator
KP	KC pump
KPH	KC preheater
KR	KC regenerator
KSEP	KC separator
KSH	KC superheater
KT	KC turbine
LAES	liquid air energy storage
LAP	liquid air pump
LAT	liquid air tank
LCAW	low-concentration ammonia–water
LMT	low-temperature methane tank
LOT	low-temperature thermal oil tank
LPT	low-temperature propane tank
LWT	low-temperature water tank
MIX	mixer
PHES	pump hydro energy storage
RTE	round-trip efficiency
SEP	separator
TV	throttle valve
**Symbols**	
*E*	exergy (MW)
*ex*	specific exergy (kJ/kg)
*h*	specific enthalpy (kJ/kg)
*m*	mass flow rate (kg/s)
*P*	pressure (MPa)
*Q*	energy (MW)
*s*	specific entropy (kJ/kg·K)
*T*	temperature (K)
*W*	power (MW)
*x*	ammonia concentration (%)
*η*	efficiency (%)
*γ*	ratio (%)
**Streams**	
A	air
C	cooling water
K	KC working fluid
M	methane
O	thermal oil
PR	propane
WA	water
**Other Subscripts**	
CH	compression heat
EVA	evaporating
ex	exergy
imp	improvement
LIQ	liquefaction ratio
vap	vapor

## References

[1] Mei S., Wang J., Tian F., Chen L., Xue X., Lu Q., Zhou Y., Zhou X. (2015). Design and Engineering Implementation of Non-supplementary Fired Compressed Air Energy Storage System: TICC-500. Sci. China Tech. Sci..

[2] Raju M., Khaitan S.K. (2012). Modeling and Simulation of Compressed Air Storage in Caverns: A Case Study of The Huntorf Plant. Appl. Energy.

[3] Smith E. (1977). Storage of Electrical Energy Using Supercritical Liquid Air. Proc. Inst. Mech. Eng..

[4] Morgan R., Nelmes S., Gibson E., Brett G. (2015). Liquid Air Energy Storage–Analysis and First Results from a Pilot Scale Demonstration Plant. Appl. Energy.

[5] Ameel B., T’Joen C., Kerpel K.D., Jaeger P.D., Huissemune H., Belleghem M.V., Paepe M.D. (2013). Thermodynamic Analysis of Energy Storage with a Liquid Air Rankine Cycle. Appl. Therm. Eng..

[6] Sciacovelli A., Vecchi A., Ding Y. (2017). Liquid Air Energy Storage (LAES) with Packed Bed Cold Thermal Storage—From Component to System Level Performance through Dynamic Modelling. Appl. Energy.

[7] Xue X., Wang S., Zhang X., Cui C., Chen L., Zhou Y., Wang J. (2015). Thermodynamic Analysis of a Novel Liquid Air Energy Storage System. Phys. Procedia.

[8] Guizzi G.L., Manno M., Tolomei L.M., Vitali R.M. (2015). Thermodynamic Analysis of a Liquid Air Energy Storage System. Energy.

[9] Howe T.A., Pollman A.G., Gannon A.J. (2018). Operating Range for a Combined, Building-Scale Liquid Air Energy Storage and Expansion System: Energy and Exergy Analysis. Entropy.

[10] Tafone A., Romagnoli A., Li Y., Borri E., Comodi G. (2017). Techno-economic Analysis of a Liquid Air Energy Storage (LAES) for Cooling Application in Hot Climates. Energy Procedia.

[11] Li Y., Cao H., Wang S., Jin Y., Li D., Wang X., Ding Y. (2014). Load Shifting of Nuclear Power Plants Using Cryogenic Energy Storage Technology. Appl. Energy.

[12] Zhang T., Chen L., Zhang X., Mei S., Xue X., Zhou Y. (2018). Thermodynamic Analysis of a Novel Hybrid Air Energy Storage System based on the Utilization of LNG Cold Energy. Energy.

[13] Antonelli M., Barsali S., Desideri U., Giglioli R., Paganucci F., Pasini G. (2017). Liquid Air Energy Storage: Potential and Challenges of Hybrid Power Plants. Appl. Energy.

[14] Al-Zareer M., Dincer I., Rosen M.A. (2017). Analysis and Assessment of Novel Liquid Air Energy Storage System with District Heating and Cooling Capabilities. Energy.

[15] She X., Peng X., Nie B., Leng G., Zhang X., Weng L., Tong L., Zheng L., Wang L., Ding Y. (2017). Enhancement of Round-Trip Efficiency of Liquid Air Energy Storage through Effective Utilization of Heat of Compression. Appl. Energy.

[16] Peng X., She X., Cong L., Zhang T., Li C., Li Y., Wang L., Tong L., Ding Y. (2018). Thermodynamic Study on the Effect of Cold and Heat Recovery on Performance of Liquid Air Energy Storage. Appl. Energy.

[17] Tafone A., Borri E., Comodi G., Broek M., Romagnoli A. (2017). Preliminary Assessment of Waste Heat Recovery Solution (ORC) to Enhance the Performance of Liquid Air Energy Storage System. Energy Procedia.

[18] Tafone A., Borri E., Comodi G., Broek M., Romagnoli A. (2018). Liquid Air Energy Storage Performance Enhancement by Means of Organic Rankine Cycle and Absorption Chiller. Appl. Energy.

[19] Zhang X., He M., Zhang Y. (2012). A Review of Research on the Kalina Cycle. Renew. Sustain. Energy Rev..

[20] Wang J., Dai Y., Gao L. (2009). Exergy Analyses and Parametric Optimizations for Different Cogeneration Power Plants in Cement Industry. Appl. Energy.

[21] Sun F., Zhou W., Ikegami Y., Nakagami K., Su X. (2014). Energy-exergy Analysis and Optimization of the Solar-boosted Kalina Cycle System 11 (KCS-11). Renew. Energy.

[22] Wang J., Yan Z., Zhou E., Dai Y. (2013). Parametric Analysis and Optimization of a Kalina Cycle Driven by Solar Energy. Appl. Therm. Eng..

[23] Pan Z., Wang J., Dai Y. (2015). Thermodynamic analysis of an integrated energy system based on compressed air energy storage (CAES) system and Kalina cycle. Energy Convers. Manag..

[24] Li R., Wang H., Yao E., Zhang S. (2017). Thermo-Economic Comparison and Parametric Optimizations among Two Compressed Air Energy Storage System Based on Kalina Cycle and ORC. Energies.

